# Evolution of Public Opinion on COVID-19 Vaccination in Japan: Large-Scale Twitter Data Analysis

**DOI:** 10.2196/41928

**Published:** 2022-12-22

**Authors:** Ryota Kobayashi, Yuka Takedomi, Yuri Nakayama, Towa Suda, Takeaki Uno, Takako Hashimoto, Masashi Toyoda, Naoki Yoshinaga, Masaru Kitsuregawa, Luis E C Rocha

**Affiliations:** 1 Graduate School of Frontier Sciences The University of Tokyo Kashiwa Japan; 2 Mathematics and Informatics Center The University of Tokyo Tokyo Japan; 3 PRESTO Japan Science and Technology Agency Kawaguchi Japan; 4 Principles of Informatics Research Division National Institute of Informatics Tokyo Japan; 5 Faculty of Commerce and Economics Chiba University of Commerce Ichikawa Japan; 6 Institute of Industrial Science The University of Tokyo Tokyo Japan; 7 National Institute of Informatics Tokyo Japan; 8 Department of Economics Ghent University Ghent Belgium; 9 Department of Physics and Astronomy Ghent University Ghent Belgium

**Keywords:** COVID-19, vaccine, vaccination, Twitter, public opinion, topic modeling, longitudinal study, topic dynamics, social events, interrupted time series regression

## Abstract

**Background:**

Vaccines are promising tools to control the spread of COVID-19. An effective vaccination campaign requires government policies and community engagement, sharing experiences for social support, and voicing concerns about vaccine safety and efficiency. The increasing use of online social platforms allows us to trace large-scale communication and infer public opinion in real time.

**Objective:**

This study aimed to identify the main themes in COVID-19 vaccine-related discussions on Twitter in Japan and track how the popularity of the tweeted themes evolved during the vaccination campaign. Furthermore, we aimed to understand the impact of critical social events on the popularity of the themes.

**Methods:**

We collected more than 100 million vaccine-related tweets written in Japanese and posted by 8 million users (approximately 6.4% of the Japanese population) from January 1 to October 31, 2021. We used Latent Dirichlet Allocation to perform automated topic modeling of tweet text during the vaccination campaign. In addition, we performed an interrupted time series regression analysis to evaluate the impact of 4 critical social events on public opinion.

**Results:**

We identified 15 topics grouped into the following 4 themes: (1) personal issue, (2) breaking news, (3) politics, and (4) conspiracy and humor. The evolution of the popularity of themes revealed a shift in public opinion, with initial sharing of attention over personal issues (individual aspect), collecting information from news (knowledge acquisition), and government criticism to focusing on personal issues. Our analysis showed that the Tokyo Olympic Games affected public opinion more than other critical events but not the course of vaccination. Public opinion about politics was significantly affected by various social events, positively shifting attention in the early stages of the vaccination campaign and negatively shifting attention later.

**Conclusions:**

This study showed a striking shift in public interest in Japan, with users splitting their attention over various themes early in the vaccination campaign and then focusing only on personal issues, as trust in vaccines and policies increased. An interrupted time series regression analysis showed that the vaccination rollout to the general population (under 65 years) increased the popularity of tweets about practical advice and personal vaccination experience, and the Tokyo Olympic Games disrupted public opinion but not the course of the vaccination campaign. The methodology developed here allowed us to monitor the evolution of public opinion and evaluate the impact of social events on public opinion, using large-scale Twitter data.

## Introduction

Vaccination is an effective mechanism to reduce the numbers of hospitalizations and deaths associated with the emergent coronavirus disease (COVID-19). With the advent of efficient vaccines after the first wave of the COVID-19 pandemic, public health efforts moved to strategies to cost-effectively immunize the population to increase survival and resume economic activity. Dose availability and uptake rates are fundamental to reaching sufficient vaccination coverage, but those numbers vary across countries in the current pandemic. One particular concern was the hesitancy regarding the safety and effectiveness of COVID-19 vaccines [1], which affected individuals’ willingness to get vaccinated in not only low- and middle-income countries [2,3], but also high-income countries [4-7]. Japan stood out among developed economies as having one of the lowest vaccine confidence levels in the population [8]. This resulted from safety concerns about the human papillomavirus (HPV) vaccine that emerged in the early 2010s as a result of misinformation spread on the adverse effects of the HPV vaccine [9,10], prompting the Japanese Ministry of Health, Labour and Welfare to suspend the proactive recommendation of the HPV vaccine from June 2013 until November 2021. Such low public confidence delayed the start of mass vaccination against COVID-19, and Japan was 2 months behind the United States, China, and European countries, leading to safety concerns and inquiries regarding the Tokyo Olympic Games that had already been postponed to August 2021. Although mass vaccination started late in Japan, the country achieved high vaccination coverage in a short time and had one of the highest vaccination rates in the world (ranking 14th among 229 countries). Japan achieved a full vaccination rate of 72.4% on October 31, 2021 [11], ranking ahead of early adopters, such as the United Kingdom (67.0%), Germany (66.2%), and the United States (58.6%).

It is unclear how public opinion affected government policies that were also influenced by the domestic economic slowdown and the concerns about the Tokyo Olympic Games. Public opinion typically reacts to policies and might serve as a barometer of government strategies. Monitoring public opinion, however, is challenging. The largest study of vaccination intention in Japan surveyed 30,000 participants [7] and found that a large proportion of the population was unsure (33%) or unwilling (11%) to receive the COVID-19 vaccine, with side effects and safety being the main reasons. Classic survey studies like this are costly, relatively slow, and, with few exceptions [8], cannot trace changes in public opinion in real time [2,6,7,12]. Large-scale studies aiming to increase accuracy and the spatiotemporal resolution of responses require advanced survey techniques. In recent years, human activity has been increasingly mediated by digital devices, leaving footprints that can be exploited to assess the population’s health and opinions [13-17]. In the context of COVID-19, social media data have been used to predict the number of new cases (incidence) [18,19] and to interpret the public perception of the pandemic [20]. Twitter has been particularly useful to monitor public opinion because users engage and react timely to environmental changes, for example, reacting to epidemic outbreaks [21,22], expressing concerns about the disease [23], accepting the pandemic situation [24], and reacting to vaccination issues [25-27]. Twitter is widely used in Japan, where more than 60% of people below 40 years old are actively engaged [28]. The pervasiveness of Twitter provides a unique source of data to monitor the evolution of public opinion during the various stages of the Japanese vaccination campaign.

In line with previous studies [25,26], we assumed that Twitter activity is a barometer of the public perception of COVID-19 vaccination. We thus focused on quantifying the public perception during the mass vaccination campaign in Japan by analyzing more than 100 million vaccine-related tweets posted by over 8 million users (approximately 6.4% of the Japanese population). The main goal was to understand the dynamics of public opinion during the vaccination campaign in Japan, which initially delayed the rollout of vaccines compared with other high-income countries. We hypothesized that such major social disruptions would lead the population to focus the debate on a few topics directly related to their daily experiences, in particular, their personal experiences with the vaccines. This debate could potentially generate social support and confidence to engage more people in the vaccination campaign. To examine the hypothesis, we identified the main topics on Twitter using the Latent Dirichlet Allocation (LDA) model [29]. We also hypothesized that public opinion would timely and semantically react to critical social events. The reactions would be for not only the stages of the vaccination campaign, but also major sports events like the Tokyo Olympic Games taking place during the vaccine rollout and the 5th COVID-19 wave. To examine the hypothesis, we quantified the effect of these critical events on the content of the debates on Twitter, using interrupted time series analysis [30].

## Methods

### Data Collection

We downloaded all Japanese tweets with the word “waku-chin” (vaccine in Japanese) posted between January 1, 2021, and October 31, 2021. The data set was provided by the NTT DATA Corporation [31]. We used data made available by NTT DATA Corporation to analyze all the vaccination-related tweets. The Twitter application programming interface (API) has a limit for the number of tweets that can be downloaded in a month. The study period was chosen to include a short period before the launch of the vaccination campaign in Japan (February 17, 2021) and a short period after the end of the Tokyo Olympic Games when the full vaccination rate reached 70% of the Japanese population (October 25, 2021). The data set contained 114,357,691 tweets. We further collected data on the tweet text, the time stamp (posting time), and whether the tweet was an original tweet or a retweet. Using data from Our World in Data [32], we obtained the daily incidence (number of new cases) of COVID-19 and the full vaccination rate (the percentage of the population who received the second dose of the COVID-19 vaccine) in Japan [11]. The COVID-19 vaccines available in Japan were Pfizer, Moderna, AstraZeneca, and Takeda (Novavax), all of which require 2 doses.

### Data Processing

Data processing and analysis were performed using Python software, version 3.9.7 (Python Software Foundation). We first extracted the plain text from the remaining tweets and removed emojis. Afterward, we segmented each text into Japanese words using the morphological analyzer MeCab [33] and removed stop words that have little analytic value (eg, “kore,” “sore,” and “suru” meaning “this,” “it,” and “do,” respectively, in Japanese). Finally, we changed words to their root forms (eg “boku” to “watashi” [“I” in Japanese] or “Utta” to “Utsu” [“inject” in Japanese]). This normalization corresponds to, for example, “viruses” to “virus” or “went” to “go” in English.

### Topic Modeling

The LDA model [29] implemented in the *Gensim* Python package [34] was used to identify topics in the Twitter data. Before the topic modeling analysis, we removed rare words, that is, words appearing in fewer than 1000 tweets that corresponded to 0.0004% of the tweets, and the most frequent words “waku-chin” (vaccine) and “sessyu” (vaccination). In addition, we identified “bot” tweets by reading typical tweets in each topic obtained by the LDA model and removed the bot tweets until the LDA model did not identify artificial topics due to bots. To determine the number of topics, we calculated the topic coherence score *C_V_* [35], which quantifies the quality of the topics obtained by the LDA model based on the probability distribution of the words. The coherence score *C_V_* is defined as a complex function of the joint probability distribution of the words [35], and a high coherence score indicates that the topics are highly interpretable for humans, that is, the subject of the tweets within a topic is likely the same. We adopted the number of topics with the highest coherence score: *K*=15 (Multimedia Appendix 1). Finally, we assigned each tweet to a topic with the highest posterior probability that was calculated based on all the words in the tweet.

### Interrupted Time Series Regression

**Figure 1:**
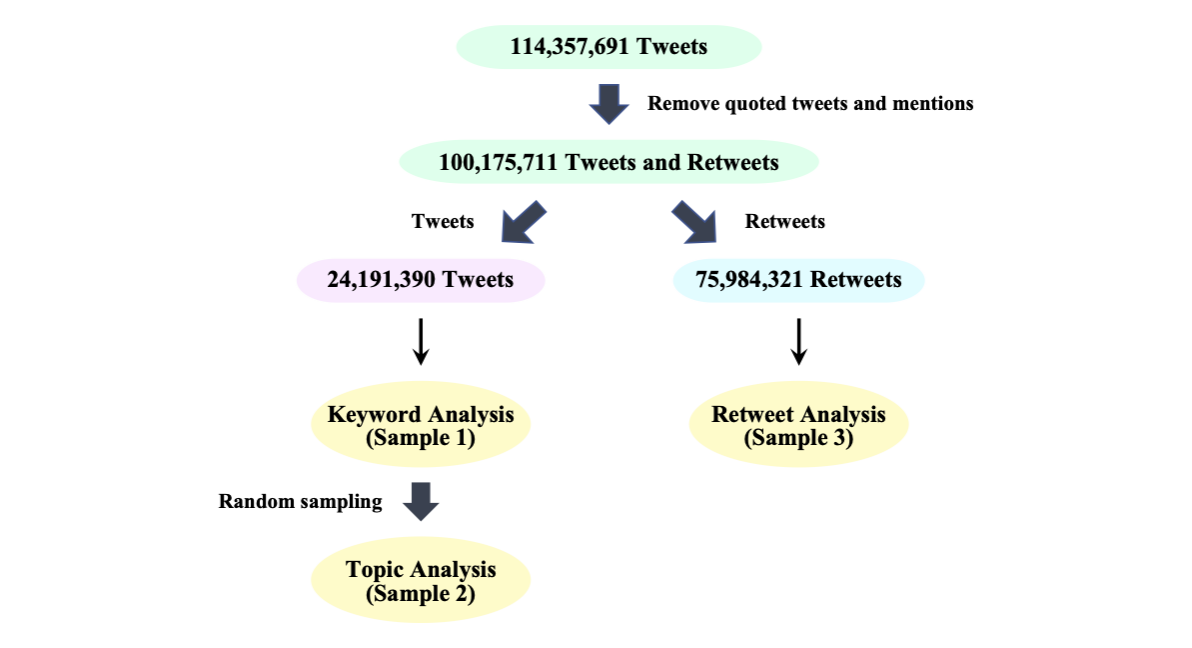
Data processing workflow.

We used interrupted time series regression [30] to quantify the impact of major events (eg, the start of the Olympic games) on the popularity of a theme in tweets. The *statsmodels* Python package [36] was used for this analysis. The theme was defined based on a subset of keywords (Multimedia Appendix 2), and the analysis was based on all the tweet data (sample 1: 24 million tweets in Figure 1). We assumed the following regression model:

*Y_t_* = *β*_0_ + *β*_1_*T* + *β*_2_*X_t_* + *β*_3_*TX_t_* + *u_t_* , **(1)**

where *Y_t_* is the percentage of a theme in tweets at time *t* (day), *T* is the number of days from the start of the observation period (*T*=0, which represents 30 days before each event), *X_t_* is a dummy variable that equals to 0 and 1 before and after the event, respectively, and *u_t_* is the error term. Here, *β*_0_ represents the baseline popularity (in percentage) at *T*=0, *β*_1_ represents the slope before the event, and *β*_2_ and *β*_3_ represent the level and slope change after the event, respectively.

A time series often exhibits autocorrelation, that is, the error terms are correlated over time, whereas the regression analysis assumes that the error terms *u_t_* are uncorrelated. To evaluate the confidence intervals of the estimated parameters, we calculated the Newey-West standard error [37,38], also known as the heteroskedasticity- and autocorrelation-consistent standard error, which is robust to the autocorrelation. We also calculated the Newey-West standard error to evaluate the confidence intervals of the linear regression analysis.

## Results

### Dataset

The original data set contained 114,357,691 vaccine-related tweets written in Japanese from January 1 to October 31, 2021. Our analysis is based on 3 samples containing either the original tweets (24,191,390/114,357,691, 21.2%) or retweets (75,984,321/114,357,691, 66.4%) that do not contain any comments (Figure 1). Quoted tweets, that is, retweets with comments (5,765,735/114,357,691, 5.0%) and mentioned tweets (8,416,245/114,357,691, 7.4%) were excluded from our analysis because they were much fewer than the tweets and retweets. The first sample (Sample 1) contained 24,191,390 tweets posted by 6,034,435 users and was used to study the evolution of public opinion, including disruptions due to critical events. A random sample of the original data (Sample 2, N=1,000,000) was then used to identify the main topics and themes, and a sample of all retweets (Sample 3) was used to study the spread of opinions.

### Vaccine-Related Tweets

**Figure 2:**
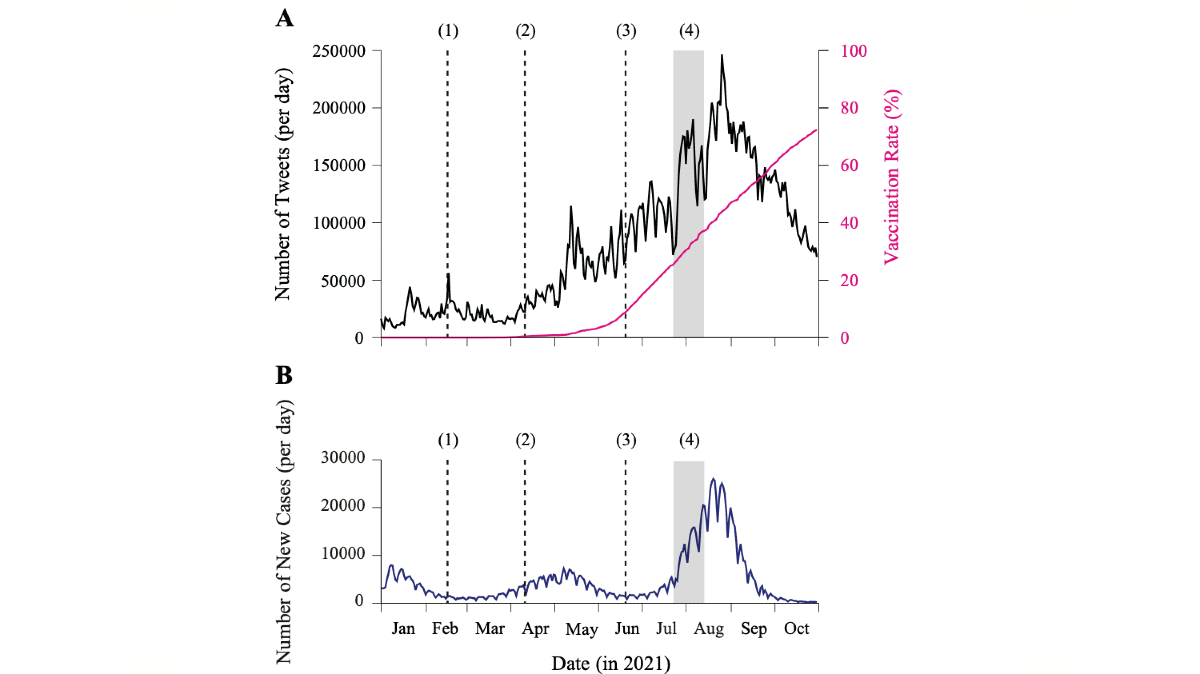
Vaccine-related tweets, vaccination rates, and incidence of COVID-19. (A) The number of vaccine-related tweets per day written in Japanese (black, left y-axis) and the fraction of the fully vaccinated population in Japan (magenta, right y-axis) between January 1 and October 31, 2021. (B) Daily incidence of COVID-19 in Japan. The vertical lines indicate 4 main events during the study period: (1) the launch of the COVID-19 vaccination campaign for essential workers; (2) the launch of vaccination for the elderly population (above 65 years old); (3) the launch of vaccination for the general population (under 65 years old); and (4) the period of the Tokyo Olympic Games.

Figure 2A shows the number of vaccine-related tweets per day during the study period and highlights the following 4 critical events [39]: (1) the launch of the COVID-19 vaccination campaign by the Japanese government on February 17, 2021, focusing initially on essential workers (eg, health care workers); (2) the start of vaccination of the elderly population (above 65 years old) on April 12, 2021; (3) the start of general public vaccination on June 21, 2021; and (4) the Tokyo Olympic Games taking place from July 23 to August 8, 2021. The first peak occurred on January 21, 2021, when Prime Minister Yoshihide Suga made a statement that “high coverage of vaccination is not a precondition for holding the Olympics in Tokyo” and the Ministry of Health, Labour and Welfare signed a contract with Pfizer Inc to supply a total of 72 million doses of its COVID-19 vaccine. Although a spike was observed at the very start of the vaccination campaign (event 1), vaccine-related tweets started to increase after event 2, when the vaccination of nonessential workers began. This coincided with the outbreak of the 4th wave in Japan (early April 2021; Figure 2B) and was followed by increased interest during the peak of infections in the 4th wave (May 13, 2021), when the online booking of vaccine appointments was launched but became overwhelmed, leaving many people without a vaccination slot. There was a sharp relative decrease in tweets at the start and end of the Olympic Games, followed by the largest peak on August 26, 2021, when a contamination scandal (approximately 1.6 million doses of the Moderna vaccine were discarded) was publicized. This last peak also coincided with the peak of the 5th wave and was followed by a substantial decrease in vaccine-related tweets, likely because of the high vaccination rate in the population (Figure 2). We found a low to moderate correlation between the number of tweets and the number of new COVID-19 cases (Spearman rank correlation: 0.322), suggesting that the effect of the pandemic situation on public opinion was not strong.

### Clustering Vaccine-Related Tweets

The ranking of the most used words on vaccine-related tweets (Sample 2 in Figure 1) revealed that 242,627 (24.3%) of them explicitly contained the word “COVID-19” (see Multimedia Appendix 3 for the frequent words). While the prevalence of specific words in tweets can reveal patterns of popular words, this measure is unable to unveil hidden semantic relations among tweets. We thus applied a machine learning methodology, the LDA model, to a sample of 1,000,000 tweets (100,000 per month, Sample 2) to automatically identify and classify (ie, cluster) tweets into meaningful topics. This monthly sampling was used here to remove the nonstationarity of the tweet activity given the imbalance in the number of vaccine-related tweets during the study period. Using LDA, we automatically identified 15 topics from the tweets (solely based on the textual content) and manually grouped them into the following 4 general themes: (1) personal issue, (2) breaking news, (3) politics, and (4) conspiracy and humor. Table 1 shows examples of representative tweets and the most popular words in each topic. Contributing terms were manually extracted from the top 30 weighted terms in the LDA model (Multimedia Appendix 4).

The most popular theme that emerged from the topic analysis was *personal issue* (Theme 1; 493,296/989,339 tweets, 49.9%), and it was formed by 2 topics about personal issues before being vaccinated, that is, personal view on vaccination and personal schedule of vaccination, and 4 topics about personal experiences after being vaccinated, that is, 1 topic about live reporting on the vaccination experience (eg, waiting room or to/from the vaccination center) and 3 topics about individual vaccination experiences including (1) complaints about discomfort, and side effects and personal life after vaccination; (2) reporting body temperature after taking the vaccine; and (3) advice to overcome side effects (Table 1).

The second most popular theme was *breaking news* (Theme 2; 210,550/989,339 tweets, 21.3%), and it included 2 topics about news on COVID-19 vaccines, such as vaccine development and approval, and vaccine effectiveness. The first topic included tweets about the development of Moderna, AstraZeneca, and Pfizer vaccines (clinical trials and government approvals) in Japan and other countries. The second topic was about the effectiveness of vaccines and contained information about mRNA vaccines, the effectiveness of vaccines against new variants, and serious side effects (eg, thrombus) of the AstraZeneca vaccine. The last topic was about booking an appointment for vaccination, in particular, about availability and whether users could successfully book a timeslot (Table 1).

*Politics* was the third most popular theme (Theme 3; 169,663/989,339 tweets, 17.1%), with 3 topics. The first topic was related to opinions on the government. For instance, users complained that the vaccination schedule in Japan was behind other countries and disagreed on holding the Tokyo Olympic Games given the low vaccination coverage. Opinions on mass media, such as complaints about unreliable information from the media and the attitude of the press inciting unrest, formed the second topic. Finally, the vaccination policy, including casual chats, for example, tweets mentioning the assignment of Mr Taro Kono (a politician famous among the young population) as vaccine minister, formed the third topic (Table 1).

**Table 1:** Topics identified from vaccine-related tweets before and during the COVID-19 vaccination campaign in Japan.

Themes and topics			Tweets (N=989,339), n (%)		Top terms contributing to the topic model		Representative tweet^a^
**Theme 1: Personal issue**			493,296 (49.9)				
	Personal view on vaccination	170,095 (17.2)		I, think, myself, scary, absolutely, feeling, alright		“I’ll be vaccinated because I want to. If you don’t want to, you don’t have to. I don’t think I should tell others to be vaccinated!”	
	Personal schedule of vaccination	57,763 (5.8)		tomorrow, today, finish, clinic, appointment, next week, this week		“I’m finally getting the Pfizer COVID-19 vaccine tomorrow. I’m so excited.”	
	Live reports of before/after vaccination	31,952 (3.2)		pain, go back, venue, swell, 30 minutes, sleepy, wait		“I’ve arrived the vaccination venue too early. I’m waiting and killing time 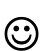 ”	
	Journal about vaccination experience	132,843 (13.4)		second time, adverse reaction, first time, yesterday, side effects, work, fine, temperature		“I’ve got the second shot. I was fine after the first shot, but I don’t think I’m fine this time. Side effect will come sooner or later.”	
	Perception after vaccination	65,490 (6.6)		pain, arm, injection, left arm, feel, discomfort		“The injection was given very quickly and was not very painful. It has been five hours since the injection, and I feel a little bit of discomfort in my left arm...”	
	Preparation for vaccination	35,153 (3.6)		fever, condition, second day, prepare, lighten, better, helpful		“I’ve got the second shot of vaccine! I need to buy sports drink when I go home... and most important of all, food for the cat!”	
**Theme 2: Breaking news**			210,550 (21.3)				
	Clinical trial and use authorization	79,247 (8.0)		Pfizer, Moderna, Ministry of Health, Labour and Welfare, development, start, clinical trial, approved		“Approval by the Ministry of Health, Labour and Welfare (MHLW) of the only vaccine for new coronavirus from US pharmaceutical giant Pfizer.”	
	Effectiveness of vaccination	74,120 (7.5)		death, effectiveness, mRNA, report, research, Israel, variant		“It is reported that mRNA vaccines are effective against corona #corona #mRNA vaccine #effective.”	
	Booking vaccination appointment	57,183 (5.8)		booking, preparation, group, available, campaign, system, local government		“The unprecedented scale of Mass vaccination: What is the preparation status of local municipalities?”	
**Theme 3: Politics**			169,663 (17.1)				
	Opinion about politics	95,219 (9.6)		Japan, measures, country, impossible, government, declaration of a state of emergency, Tokyo		“To the idiots in the government: if you can inoculate corona vaccine to all Japanese citizens, you can hold Olympic and Paralympic, but if you can’t, cancel them.”	
	Opinion about mass media	41,094 (4.2)		anxiety, information, news coverage, rumor, media, explanation, fact		“The mass media raised fears with coronas, and now they are raising fears with vaccines. Media should report the facts unbiasedly instead of raising fears.”	
	Vaccination policy	33,350 (3.4)		third time, recently, tweet, video, shit, laugh		“It may be better to take a wait-and-see approach to the vaccination. It could be bad.”	
**Theme 4: Conspiracy and humor**			115,830 (11.7)				
	Population control	41,428 (4.2)		population, human being, world, cause, conspiracy theory, reduction		“They developed the corona vaccine for the purpose of the Deep State agenda: global human enslavement, depopulation and money making!”	
	Effect on the body	30,221 (3.1)		children, 5G, freedom, destruction, discrimination		“It’s very exciting to be able to connect to 5G when you are vaccinated!”	
	Internet meme	44,181 (4.5)		cine-cine		“Vac-vac-cine-cine! Vac-vac-cine-cine! Vac-vac! Cine-cine! Cine-cine vac-vac!”	

^a^Original tweets are in Japanese.

The least popular theme contained topics related to conspiracy and humor (Theme 4; 115,830/989,339 tweets, 11.7%). The first topic was about control of the population, for example, the conspiracy theory that “the purpose of COVID-19 vaccination was to reduce the global population,” and the second topic was about the effects on the body, for example, the theory that “COVID-19 vaccines are a ploy to connect people to the 5G network.” Internet memes formed the third topic, for example, the popular “Vac-vac-cine-cine” (from “vaccine vaccine” because a person needs 2 vaccine shots to be fully vaccinated and because the combination of these words sounds like “exciting” and “male genitalia” in Japanese) (Table 1).

### Evolution of the Popularity of Themes

**Figure 3:**
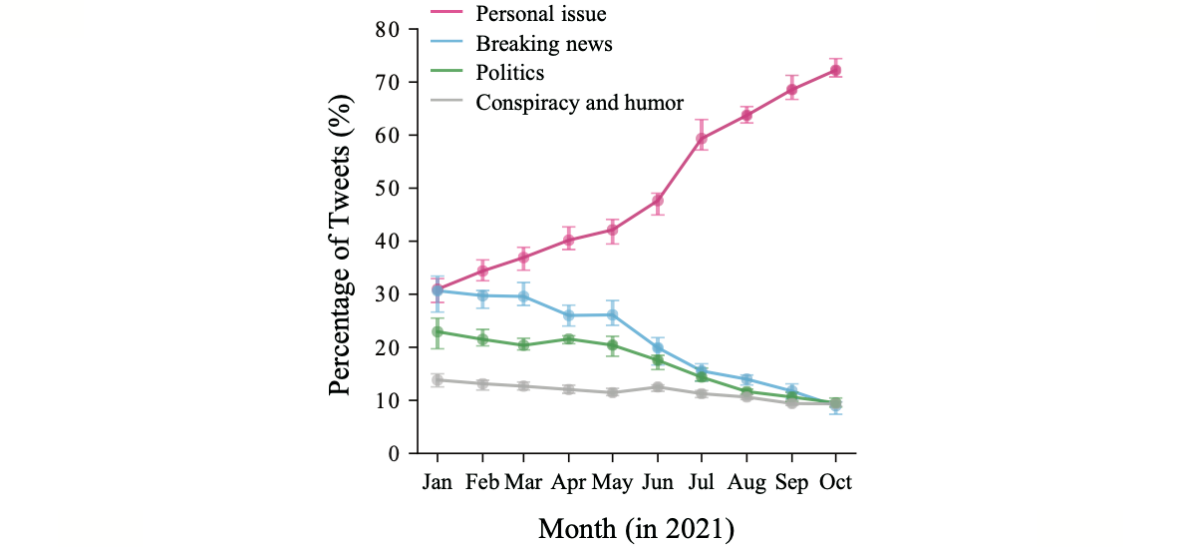
Popularity of the study themes. Each line represents the percentage of tweets in each theme (Table 1) over time. The percentage is calculated monthly from a sample of vaccine-related tweets (1 million tweets: Sample 2).

Previous research has shown that the number of tweets about a particular topic reflects the users’ attention to that topic [40,41]. We thus estimated the popularity of tweets for each topic (grouped in 4 major themes; see the previous section) to monitor temporal changes in the interest of users (Figure 3). *Personal issue* (Theme 1) continuously increased, starting at nearly 30% and increasing to over 70% by the end of the study period. *Breaking news* (Theme 2) and *politics* (Theme 3), on the other hand, declined steadily from nearly 30% and 25%, respectively, to around 10%, dropping more significantly after June, when vaccination became available for people under 65 years old (the majority of Twitter users). *Conspiracy and humor* (Theme 4) also reduced slightly during the period and overall remained relatively low. We further validated this result by creating a subset of keywords for each theme (Multimedia Appendix 2) and then extracting all tweets of each theme from the original data set (24 million tweets) (Multimedia Appendix 5). The linear regression analysis (Table 2) showed a statistically significant increase in the tweets about *personal issue* (Theme 1) and a decrease in the other themes, with *breaking news* (Theme 2) and *politics* (Theme 3) decreasing 5 times in comparison to *conspiracy and humor* (Theme 4). These trends revealed a shift in the concerns of Twitter users, who initially shared their attention over personal issues (individual aspect), collecting information from the news (knowledge acquisition), and government decisions (the course of the vaccination campaign) and then focused mostly on personal issues once the vaccination campaign was effectively implemented in the general population.

**Figure 4:**
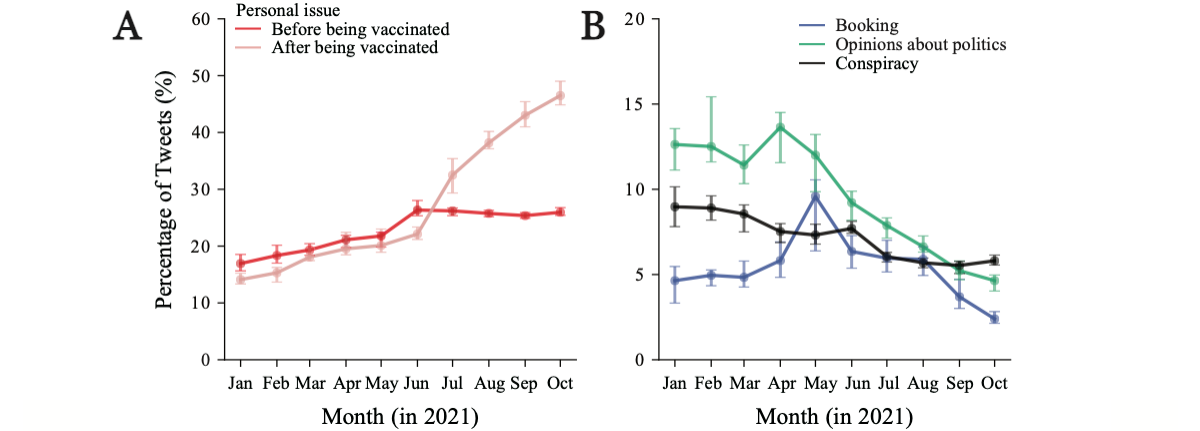
Popularity of the topics. Each line represents the percentage of tweets over time. (A) Aggregated topics about personal issue before/after being vaccinated (Theme 1). (B) Topics about booking vaccination appointment (Booking in Theme 2), opinions about politics (Opinions about politics in Theme 3), and aggregated topics about conspiracy theories, that is, population control and effect on the body (Conspiracy in Theme 4). The percentage is calculated monthly from a sample of vaccine-related tweets (1 million tweets: Sample 2).

The evolution of specific topics reflected finer aspects of the opinion dynamics. The combined topics about personal issues before being vaccinated (ie, personal view and personal schedule) increased after May followed by a slight decrease after August (Figure 4A). This pattern reflected increasing concerns with vaccination and the Tokyo Olympic Games that ended in early August. The combined topics about a user’s experience after being vaccinated (ie, live reports, journal, perception, and preparation) showed a sharp increase after June, when the vaccination of the general population began (Figure 4A). Moreover, 17.9% (17,760/99,461) of the tweets belonged to the topic about personal issues after being vaccinated, even in January before the vaccination campaign in Japan. This is because the LDA model assigned a topic based on the words in a tweet (see Multimedia Appendix 4 for the top 30 terms).

In contrast, the popularity of conspiracy theories (population control and effect on the body) decreased steadily, indicating that education built up confidence in the vaccines (Figure 4B). Opinions on the booking of vaccination appointments peaked in May, when the booking system was launched. Opinions on politics peaked in April and then decreased substantially, reflecting an initial criticism toward the government for the late implementation of mass vaccination, followed by approval once the campaign rolled out. Again, we validated these findings by extracting the corresponding tweets using a subset of keywords for each topic or aggregated topic (Multimedia Appendix 2) and confirmed the trends (Multimedia Appendix 6), with a low prevalence of words related to conspiracy theories (1,452,528/24,032,297 tweets, 6.0%). This result also confirmed that the initial concerns about the government and the reliability of the vaccines became secondary once the vaccination reached most of the population and personal experiences became dominant.

**Table 2:** Linear regression analysis of the popularity time series of the themes extracted by keywords.

Variable			Theme^a^						
			Theme 1		Theme 2		Theme 3		Theme 4
**Intercept**									
	Coefficient	25.8^b^		44.0^b^		24.0^b^		8.75^b^	
	95% CI	23.9 to 27.6		41.2 to 46.8		22.2 to 25.8		8.22 to 9.27	
**Slope**									
	Coefficient	0.142^b^		−0.082^b^		−0.070^b^		−0.014^b^	
	95% CI	0.133 to 0.152		−0.097 to −0.067		−0.078 to −0.061		−0.017 to −0.011	

^a^Theme 1: personal issue; Theme 2: breaking news; Theme 3: politics; and Theme 4: conspiracy and humor.

^b^Statistically significant change (*P*<.05).

### Shift in Interest After Critical Events

**Figure 5:**
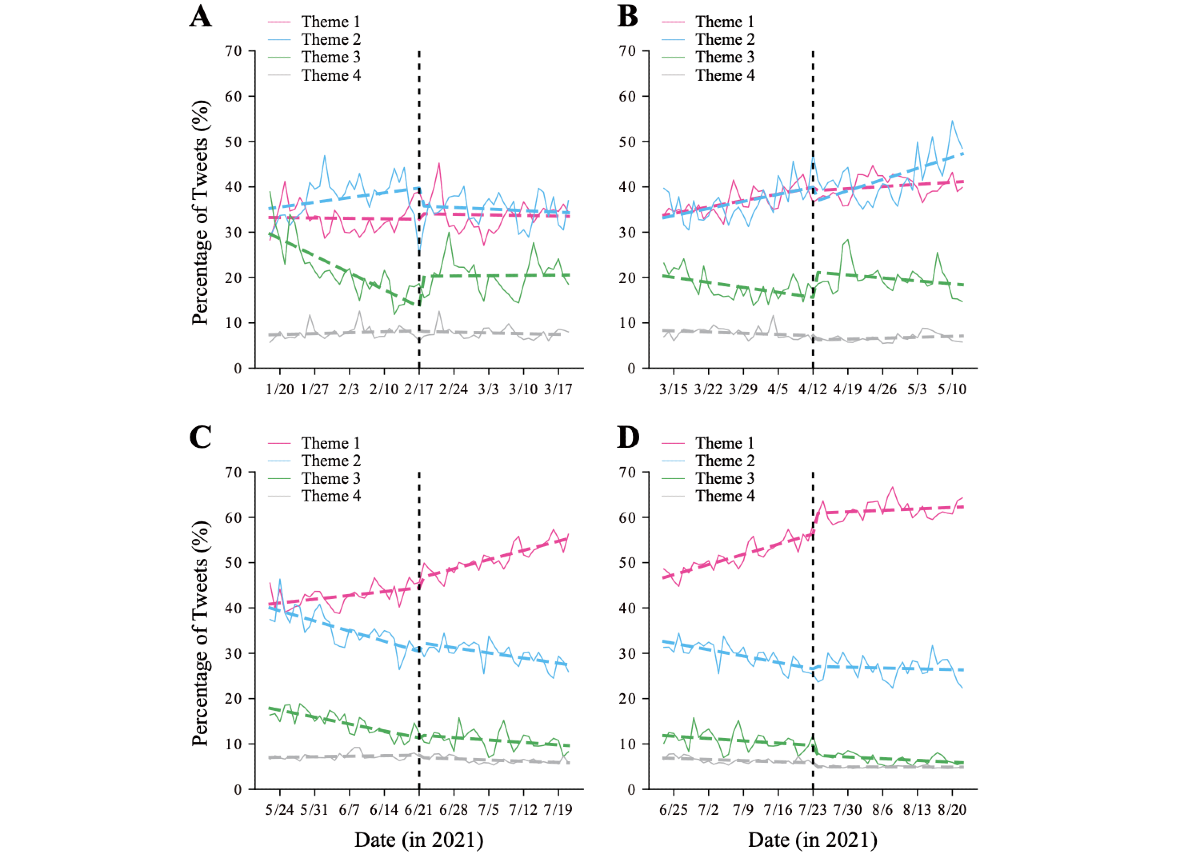
Impact of social events on the popularity of the themes. We applied interrupted time series regression to the popularity time series of each theme (Theme 1: personal issue; Theme 2: breaking news; Theme 3: politics; and Theme 4: conspiracy and humor). We examined the following 4 major events during the vaccination period: (A) vaccination start for health workers, (B) vaccination start for older people, (C) vaccination start for the general population (under 65 years), and (D) start of the Olympic Games in Tokyo.

Specific events may have social and individual consequences and may affect public opinion and discussion of different themes. Four critical events marked the vaccination campaign in Japan during 2021 (the various stages of the vaccination campaign and the Tokyo Olympic Games; Figure 2). To test our hypothesis of critical events on opinion dynamics, we performed interrupted time series regression [30] to estimate the changes in the popularity of themes (see the Methods section). We first calculated the popularity (ie, the percentage of tweets) of 4 themes defined by subsets of keywords (Multimedia Appendix 2). The themes were as follows: Theme 1, personal issue; Theme 2, breaking news; Theme 3, politics; and Theme 4, conspiracy and humor. In this analysis, the level parameter (*β*_2_ in Equation 1) indicates a shift in the relative attention, whereas the slope parameter (*β*_3_ in Equation 1) indicates a shift in the rate of popularity increase of a given theme. Table 3 shows that *politics* (Theme 3) was the theme most affected by these events. The impact of the general population vaccination rollout and the Tokyo Olympic Games on public opinion was larger than that of the other critical events, and they affected all aspects of public opinion. The vaccination of health workers positively shifted the popularity of the *politics* theme, likely because of increasing expectations of rolling out mass vaccination. The vaccination of the elderly population only positively shifted the trend. On the other hand, both the vaccination of the general population and the Tokyo Olympic Games negatively shifted the interest in politics, suggesting relatively fewer concerns with government policies. Furthermore, the vaccination rollout of the general population increased the rate of tweets about practical advice and personal experience. Finally, the start of the Tokyo Olympic Games caused an increase in interest in personal issues that remained nearly constant afterward (Figure 5), likely because of the large vaccination coverage achieved during this period.

**Table 3:** Changes in the popularity of the 4 themes at critical events.

Variable			Theme^a^			
			Theme 1	Theme 2	Theme 3	Theme 4
**Health workers**						
	**Level (*β*_2_)**					
		Coefficient	1.21	−3.93	6.74^b^	−0.14
		95% CI	−4.51 to 6.93	−8.88 to 1.03	1.27 to 12.2	−1.45 to 1.18
	**Slope (*β*_3_)**					
		Coefficient	−0.01	−0.20	0.54^b^	−0.05
		95% CI	−0.32 to 0.31	−0.55 to 0.15	0.30 to 0.80	−0.14 to 0.03
**Elderly population**						
	**Level (*β*_2_)**					
		Coefficient	−0.71	−3.25	5.59^b^	−0.98
		95% CI	−3.54 to 2.13	−7.79 to 1.28	1.60 to 9.58	−2.11 to 0.15
	**Slope (*β*_3_)**					
		Coefficient	−0.14	0.13	0.06	0.07
		95% CI	−0.28 to 0.01	−0.17 to 0.42	−0.15 to 0.27	−0.01 to 0.14
**General population**						
	**Level (*β*_2_)**					
		Coefficient	2.17 ^b^	1.95	0.60	−0.51
		95% CI	0.49 to 3.85	−0.15 to 4.05	−1.21 to 2.42	−1.33 to 0.30
	**Slope (*β*_3_)**					
		Coefficient	0.17^b^	0.16^b^	0.14^b^	−0.06^b^
		95% CI	0.05 to 0.29	0.06 to 0.26	0.06 to 0.22	−0.09 to −0.02
**Olympic Games**						
	**Level (*β*_2_)**					
		Coefficient	4.57^b^	0.54	−2.17^b^	−0.89^b^
		95% CI	2.80 to 6.35	−1.42 to 2.51	−3.19 to −1.15	−1.32 to −0.46
	**Slope (*β*_3_)**					
		Coefficient	−0.27^b^	0.18^b^	0.02	0.03^b^
		95% CI	−0.37 to −0.17	0.04 to 0.32	−0.05 to 0.08	0.00 to 0.07

^a^Theme 1: personal issue; Theme 2: breaking news; Theme 3: politics; and Theme 4: conspiracy and humor.

^b^Statistically significant change (*P*<.05).

### Spread of Opinions

**Figure 6:**
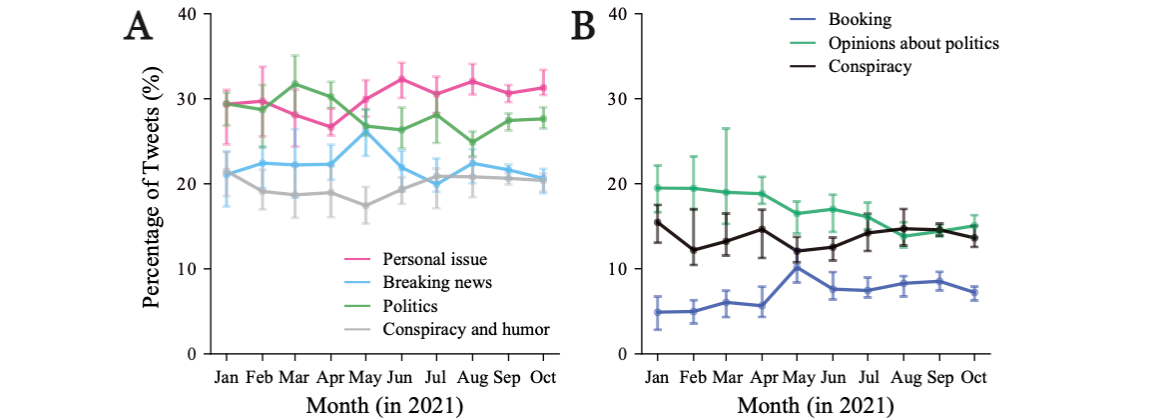
Popularity of retweeted themes or topics. Each line represents the percentage of frequently retweeted tweets (retweeted more than 10 times in a day) in each theme (A) or topic (B) over time. The topics of Conspiracy represent combined topics of population control and effect on the body.

A tweet is a unidirectional process of sharing information with the community. Retweeting, on the other hand, is a social process where users engage and share tweets to spread opinions on their own social network [42]. The analysis of 75,984,321 retweets by 3,917,181 users (Sample 3) showed a higher prevalence of retweets about *personal issue* (Theme 1) and *politics* (Theme 3) in comparison to *breaking news* (Theme 2) and *conspiracy and humor* (Theme 4) (Figure 6A). Those observations aligned with the theory of complex contagion, since users mostly engaged with tweets (by retweeting) related to personal experiences and political opinion rather than tweets sharing hard-to-verify information, such as vaccine reliability and conspiracy theories, that might have negative consequences and might affect the credibility of the user retweeting [43]. Similar to the popularity of certain topics, the social process is also intensified during certain periods (Figure 6B). For instance, the topic of booking an appointment exhibited a peak in May, coinciding with the popularity of this topic, whereas the topic of politics declined after April, when vaccination of the elderly population started.

## Discussion

### Principal Findings

This study aimed to understand the dynamics of public opinion during the vaccination campaign in Japan, which initially delayed the rollout of vaccines compared with other high-income countries. We leveraged the textual information in tweets and performed a topic analysis of vaccine-related tweets to identify 15 topics further grouped into the following 4 major themes: (1) personal issue, (2) breaking news, (3) politics, and (4) conspiracy and humor, during the vaccination campaign in Japan (from January 1 to October 31, 2021). We found a striking shift in public interest, with users splitting their attention over various themes early in the campaign and then focusing on personal issues, as trust in vaccines and policies built up with an effective vaccination campaign. Next, we examined the effect of critical social events on the popularity of the tweet themes. We found that the vaccination rollout to the general population (under 65 years old) increased the popularity of tweets about practical advice and personal vaccination experience. This result implies that the start of vaccination of the general population was a critical event for Twitter users (mostly 20-30 years old in Japan). We also found that the popularity of the themes remained at the same level during the Olympic Games.

### Comparison With Prior Work

Previous studies using social media (Twitter and Reddit) [25-27] to study public opinions of COVID-19 vaccination in different countries were limited in sample size and did not cover the whole vaccination campaign. Therefore, only topics related to breaking news [25-27] and politics [25] were identified. We showed, however, that personal issue is a common topic emerging during critical periods and is fundamental for a successful mass vaccination campaign, since it bonds people via social support. Furthermore, our findings are more robust than the findings of existing studies [25-27] because the main results (Figure 3 and 4) were confirmed by a robustness analysis using the whole data set (Multimedia Appendix 5 and Multimedia Appendix 6). While we could not collect all tweets via the Twitter API, we could still analyze all vaccination-related tweets by using comprehensive data from NTT DATA Corporation.

Furthermore, the interrupted time series regression analysis showed that the vaccination rollout of the general population and the Tokyo Olympic Games affected public opinion more than other critical events. Public opinion on politics was the most significantly affected debate, positively shifting attention early in the vaccination campaign and negatively later. In addition, social dialogue was maintained with tweets about personal issues mostly retweeted when vaccination reached the adult population, which is the most active user group on Twitter.

### Limitations

There are limitations in our study. First, it was impossible to avoid sampling bias in the online data set even though we analyzed all Japanese tweets including the word “waku-chin” (vaccine in Japanese). We analyzed the tweets posted by 8 million users (approximately 6.4% of the Japanese population), which is comprehensive and represents the opinion of active users but might not fully represent the general public. Nevertheless, Twitter data are representative of the opinion of the younger generation (20-30 years old) in Japan, which is supported by a survey [28] reporting that more than 60% of the population below 40 years old is actively engaged on Twitter. To minimize potential sampling biases, we resampled the original data of 6 million users to remove temporal effects. Unlike standard survey studies, we were unable to collect sociodemographic information and thus could not stratify the analysis to age group, location, education, and gender [3,7]. Stratification would help us to assess the extent to which certain social groups (eg, adults vs elderly) and locations (eg, Tokyo during the Olympic Games) were affected. Second, the study population was limited to those using Twitter in Japan. While this limitation enables us to understand the public opinion of Japanese Twitter users, the results may not be generalizable to other countries, such as the United States, China, and European and African countries. Future work is necessary to compare the public opinions of users in Japan to those in other countries. Finally, the inclusion criterion of the keyword “vaccine” may have captured tweets not relevant to COVID-19, such as those related to the HPV vaccine or to pet vaccination. To assess this aspect, we manually reviewed tweets and found that most of them were not contaminated by discussions of other types of vaccines.

We used the LDA model to identify topics from tweets, which assigns a tweet to a topic based on the words present. However, a topic might contain several issues. For example, more than 10% of the tweets in January (before the vaccination campaign in Japan) were classified under the topic “after being vaccinated.” This is because the terms that contributed to the topic (eg, “side reaction” and “mask”) were used in January. Moreover, we applied interrupted time series analysis to examine the impact of critical social events on popular topics on Twitter. While the standard interrupted time series analysis [30] neglected the effect of autocorrelation in the time series, we incorporated it by calculating the Newey-West standard error to evaluate the confidence intervals. Notably, the low to moderate correlation between the number of tweets and the number of new cases in a day suggests that the pandemic status might impact the popular topics on Twitter. It would be interesting to further investigate the effect of the pandemic’s status on the popular topics on Twitter and incorporate it into the analysis. Finally, we manually identified bot retweets that impacted the topics obtained by the LDA model. Bot detection is a challenging research issue, and there have been only few attempts to identify bots (eg, Botometer [44]). Although we applied Botometer to our data set, it was unable to identify the bots we excluded in this study. Further studies are required to establish guidelines to identify tweets posted by bot accounts.

### Conclusions

We studied the evolution of public opinion regarding COVID-19 vaccination in Japan by analyzing more than 100 million vaccine-related tweets. We identified the following 4 themes in the tweets: (1) personal issue, (2) breaking news, (3) politics, and (4) conspiracy and humor. We found a striking shift in public interest. Users split their attention over various themes early in the campaign and then focused on personal issues, as trust in vaccines built up with an effective vaccination campaign. An interrupted time series regression analysis showed that the vaccination rollout to the general population (under 65 years old) increased the popularity of tweets about practical advice and personal vaccination experience, and the Tokyo Olympic Games disrupted public opinion but not the course of the vaccination campaign. The methodology developed here allowed us to monitor the evolution of public opinion and evaluate the impact of social events on public opinion, using large-scale Twitter data.

## Appendix

Multimedia Appendix 1 Dependency of the number of topics on the coherence score.

Multimedia Appendix 2 List of keywords for the 4 themes and their subthemes.

Multimedia Appendix 3 Top 50 used words in vaccine-related tweets: Sample 2 (1 million tweets).

Multimedia Appendix 4 Top 30 contributing terms of each topic identified from vaccine-related tweets.

Multimedia Appendix 5 Popularity of the themes defined based on the subset of keywords (Multimedia Appendix 2). Each line represents the percentage of tweets of each theme over time. The percentage was calculated for each month (A) and day (B). Dashed lines in panel (B) represent the fitted lines obtained by the linear regression.

Multimedia Appendix 6 Popularity of the subthemes defined based on the subset of keywords (Multimedia Appendix 2). (A) Subthemes related to personal issue corresponding to Theme 1 (Figure 4A). (B) Subthemes related to reservation, politics, and conspiracy theories corresponding to Themes 2, 3, and 4, respectively (Figure 4B).

## Abbreviations

API: application programming interface
HPV: human papillomavirus
LDA: Latent Dirichlet Allocation
