# Supplementary figures and images for: Evolution of Public Opinion on COVID-19 Vaccination in Japan: Large-Scale Twitter Data Analysis
Source: J Med Internet Res. 2022 Dec 22;24(12):e41928. doi: 10.2196/41928 (PMC9856430; doi:10.2196/41928)

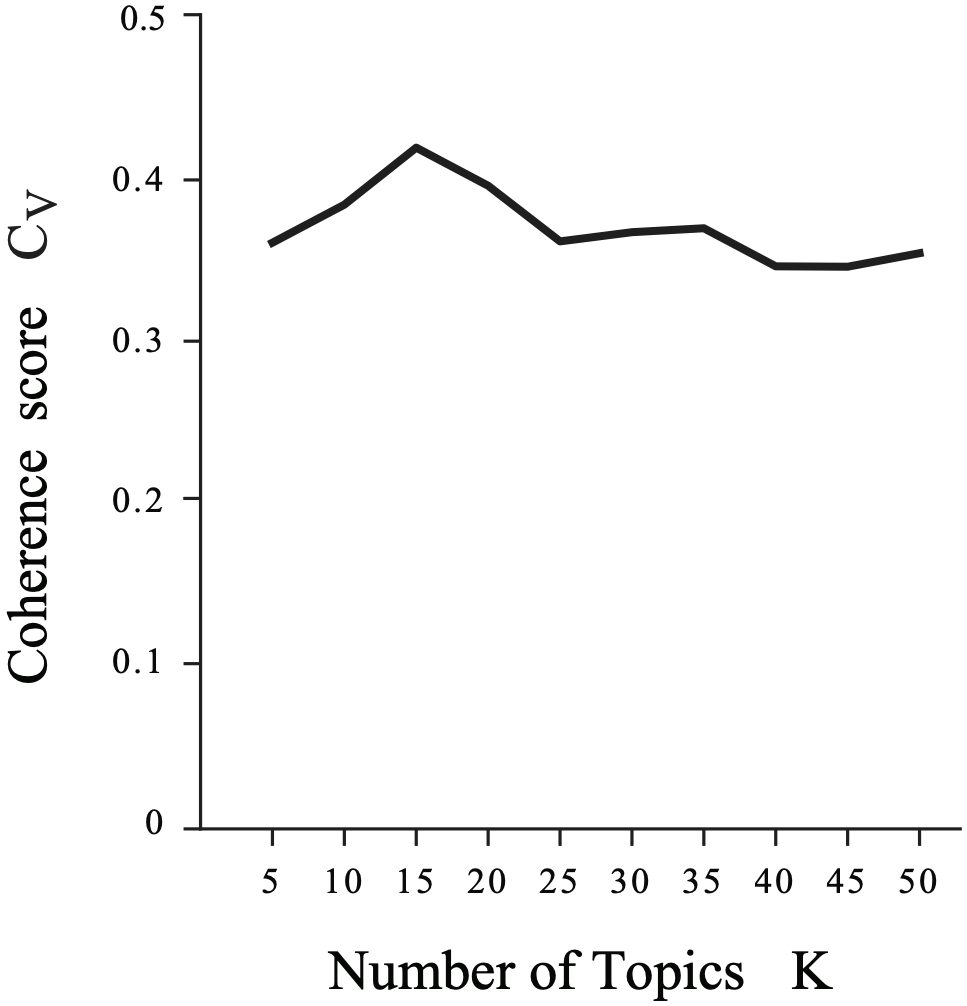

Supplement: Multimedia Appendix 1 [file jmir_v24i12e41928_app1.png]

**Multimedia Appendix 3.** Top 50 used words in vaccine-related tweets: Sample 2 (1 million tweets).

**
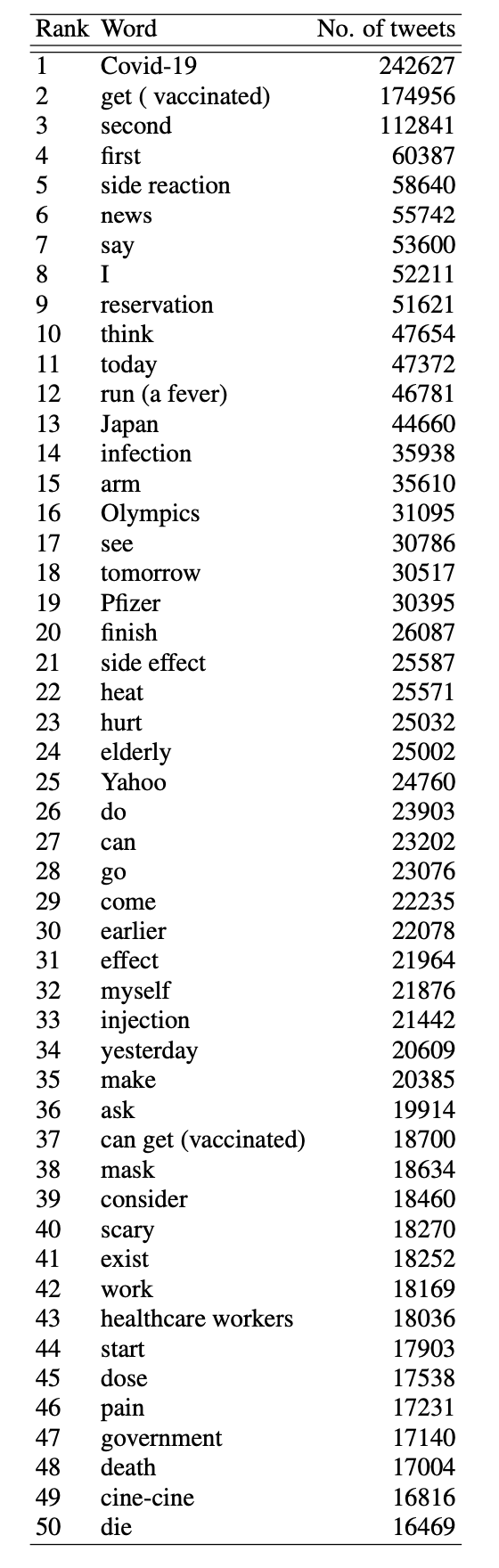
**

Supplement: Multimedia Appendix 3 [file jmir_v24i12e41928_app3.docx]

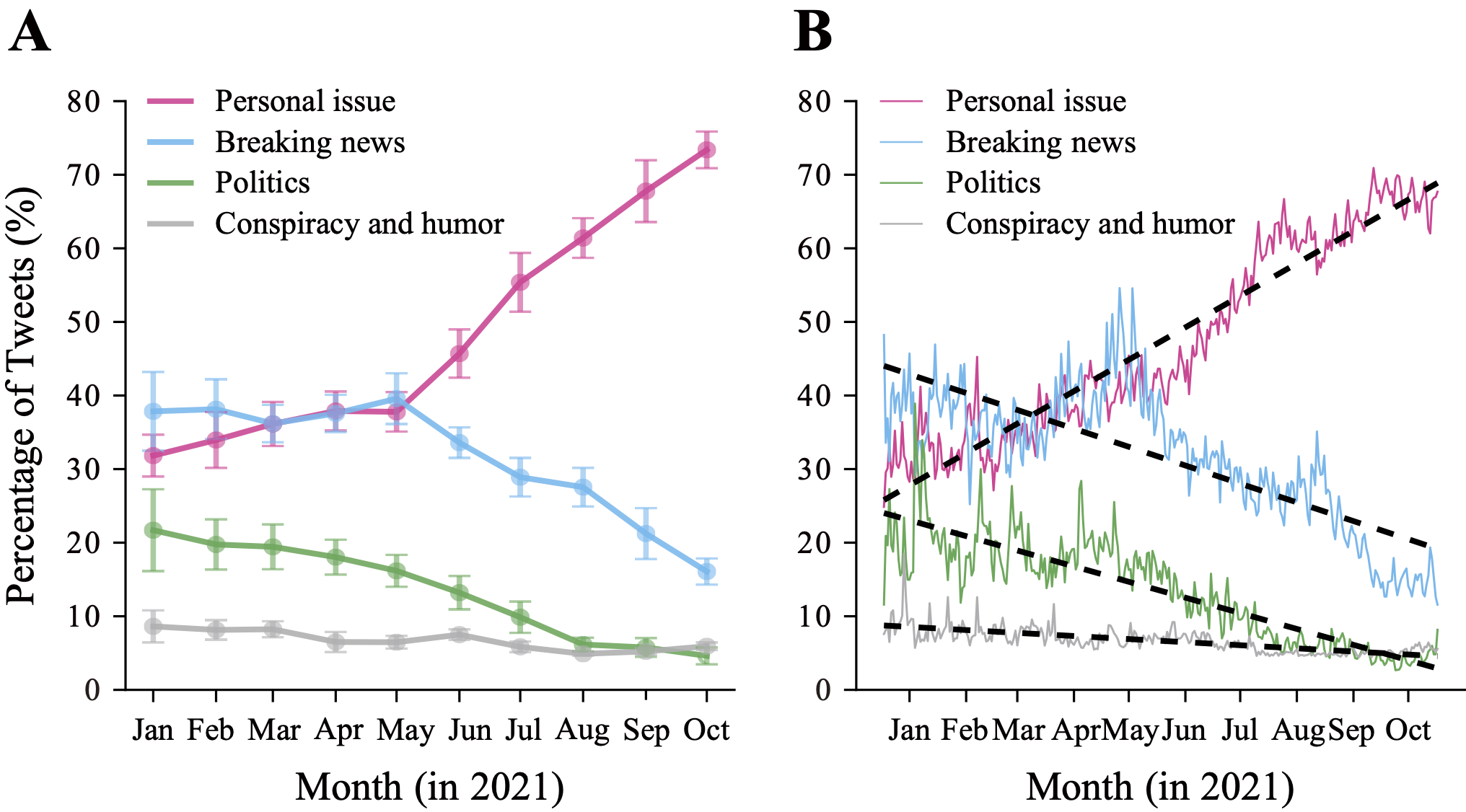

Supplement: Multimedia Appendix 5 [file jmir_v24i12e41928_app5.png]

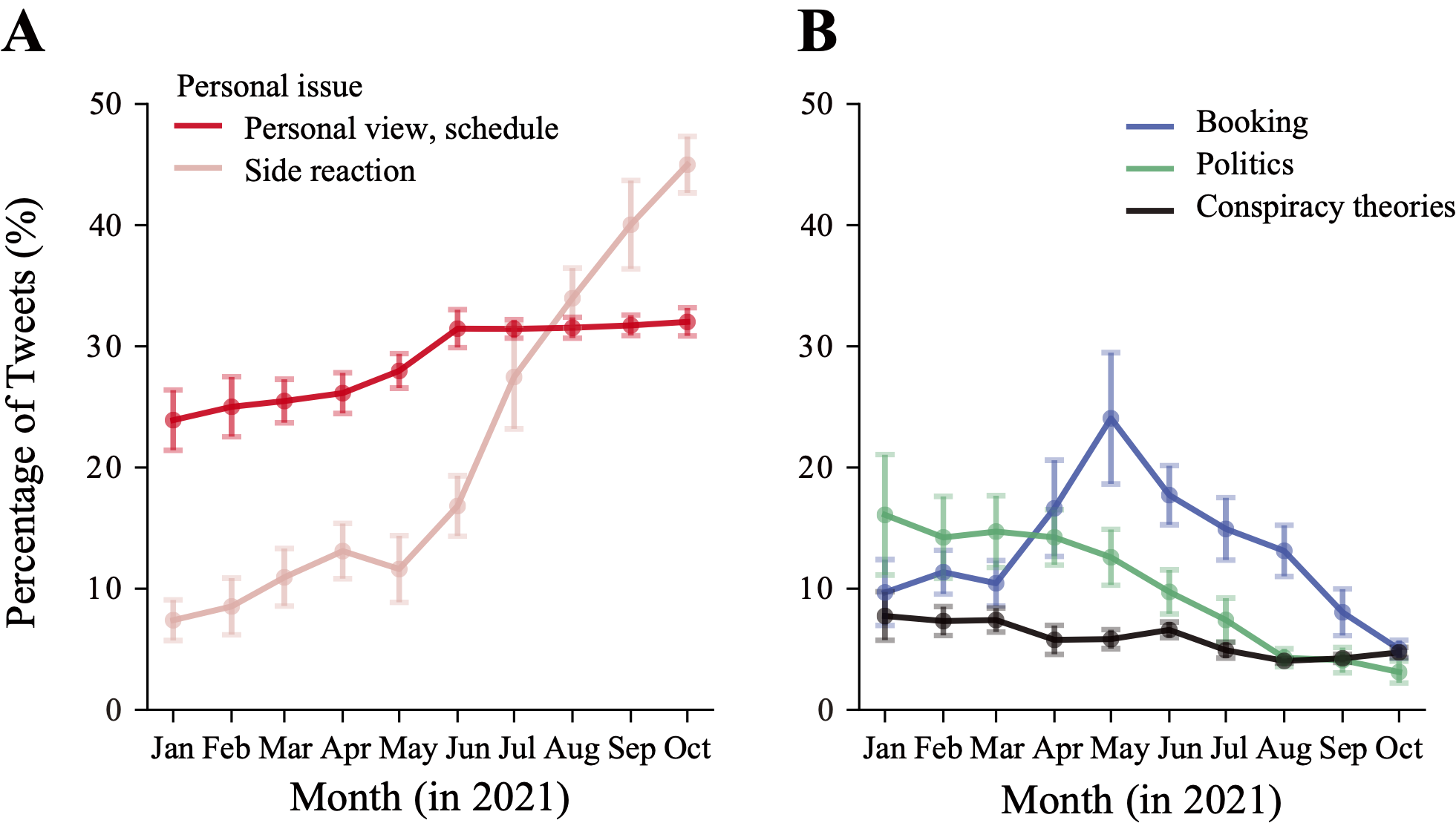

Supplement: Multimedia Appendix 6 [file jmir_v24i12e41928_app6.png]
