# Supplementary material for: Evolution of Public Opinion on COVID-19 Vaccination in Japan: Large-Scale Twitter Data Analysis
Source: J Med Internet Res. 2022 Dec 22;24(12):e41928. doi: 10.2196/41928 (PMC9856430; doi:10.2196/41928)

**Multimedia Appendix 2.** List of keywords for the 4 themes and their subthemes.

Keywords are manually assigned from the result of the topic modeling (Table 1) and reading typical tweets in each topic.


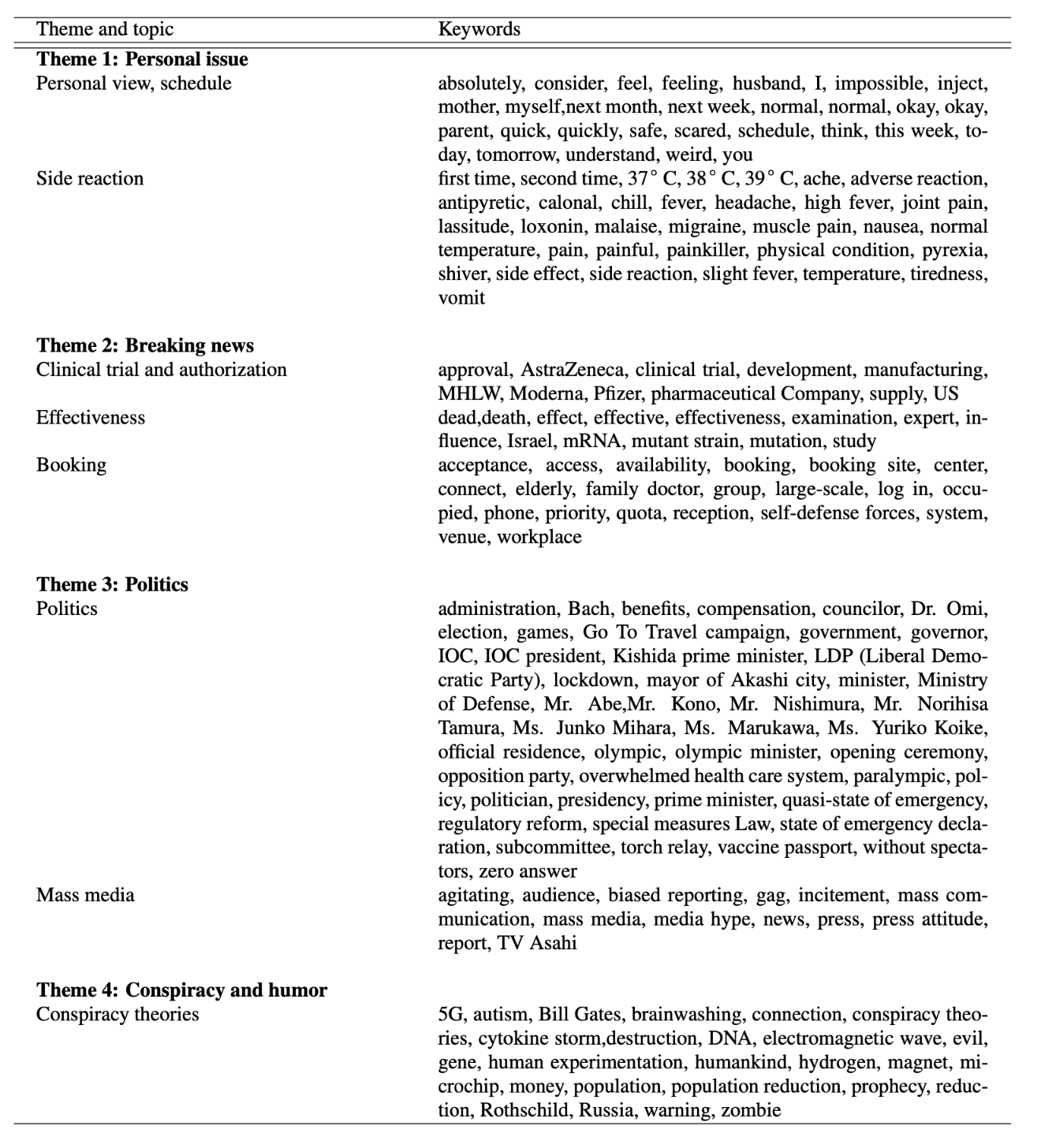

Supplement: Multimedia Appendix 2 [file jmir_v24i12e41928_app2.docx]
