# Supplementary material for: Evolution of Public Opinion on COVID-19 Vaccination in Japan: Large-Scale Twitter Data Analysis
Source: J Med Internet Res. 2022 Dec 22;24(12):e41928. doi: 10.2196/41928 (PMC9856430; doi:10.2196/41928)
Supplement: Multimedia Appendix 4 [file jmir_v24i12e41928_app4.docx]

**Multimedia Appendix 4.** Top 30 contributing terms of each topic identified from vaccine-related tweets.

**
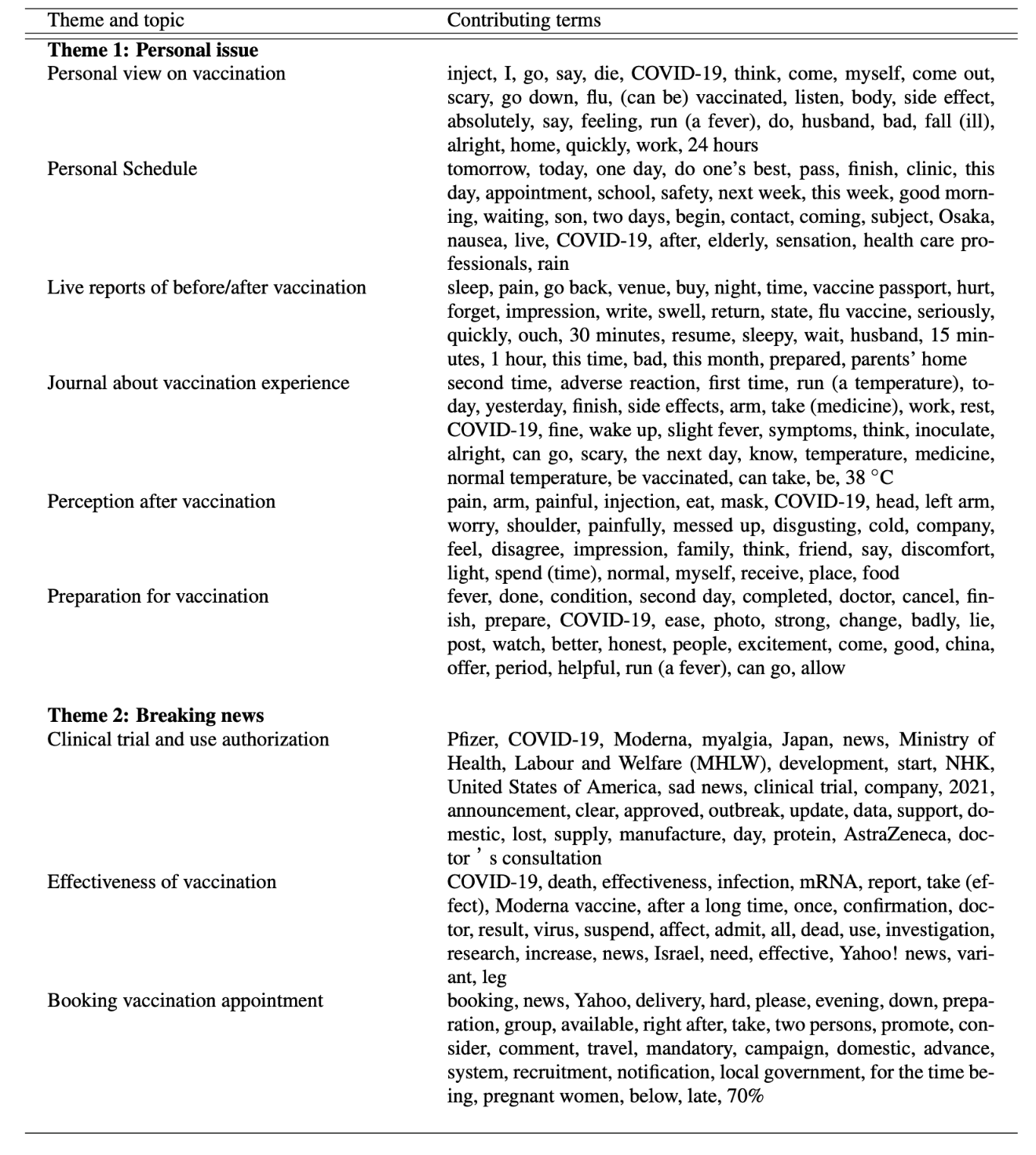
**

**
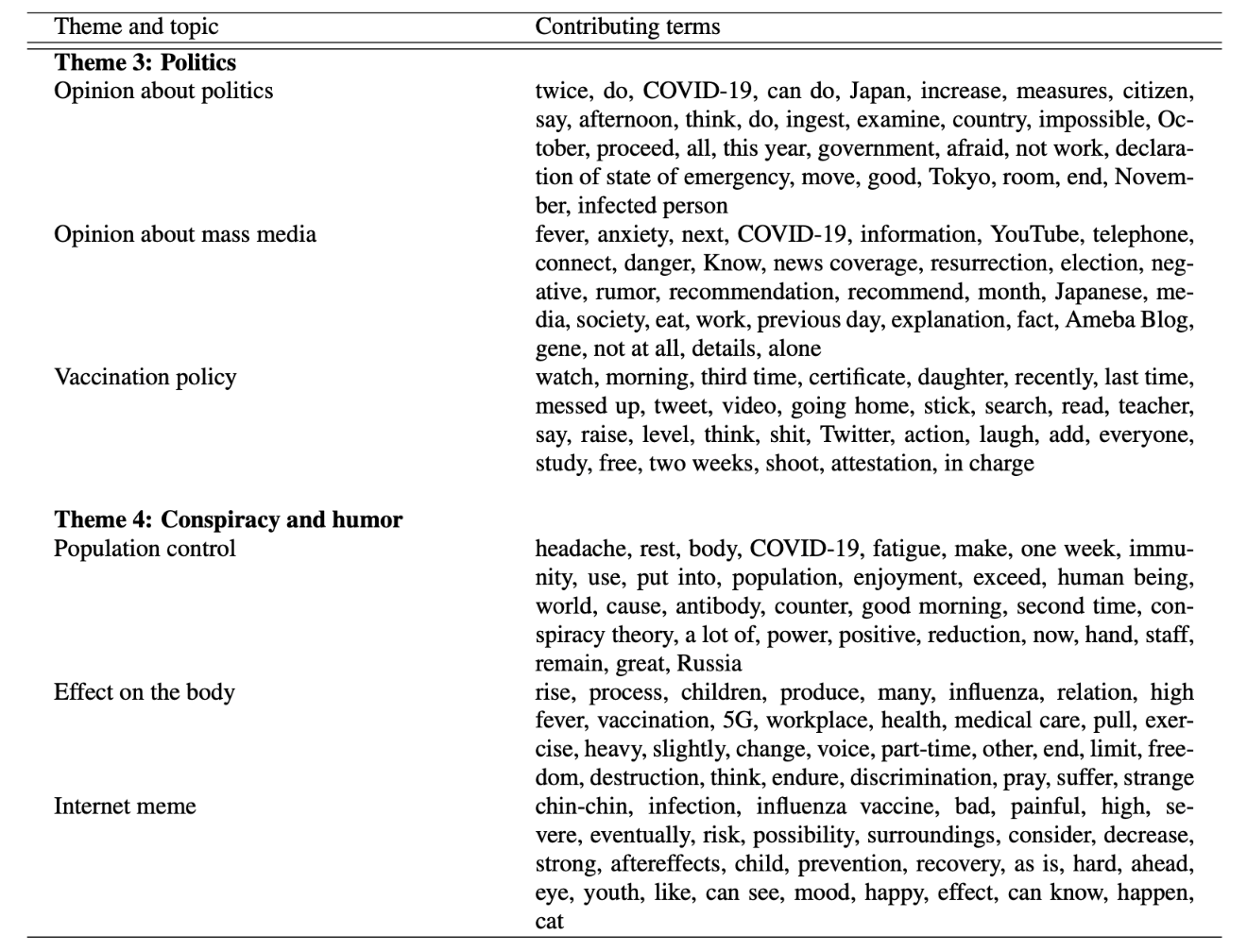
**
